# Recent advances in our understanding of brown and beige adipose tissue: the good fat that keeps you healthy

**DOI:** 10.12688/f1000research.14585.1

**Published:** 2018-07-24

**Authors:** Michael E. Symonds, Peter Aldiss, Mark Pope, Helen Budge

**Affiliations:** 1 Early Life Research Unit, Division of Child Health, Obstetrics & Gynaecology, School of Medicine, University of Nottingham, Nottingham, UK; 2 Nottingham Digestive Disease Centre and Biomedical Research Centre, School of Medicine, University of Nottingham, Nottingham, NG7 2UH, UK

**Keywords:** Brown adipose tissue

## Abstract

Brown adipose tissue (BAT) possesses a unique uncoupling protein (UCP1) which, when activated, enables the rapid generation of heat and the oxidation of lipids or glucose or both. It is present in small amounts (~15–350 mL) in adult humans. UCP1 is rapidly activated at birth and is essential in preventing hypothermia in newborns, who rapidly generate large amounts of heat through non-shivering thermogenesis. Since the “re-discovery” of BAT in adult humans about 10 years ago, there has been an exceptional amount of research interest. This has been accompanied by the establishment of beige fat, characterised as discrete areas of UCP1-containing cells dispersed within white adipocytes. Typically, the amount of UCP1 in these depots is around 10% of the amount found in classic BAT. The abundance of brown/beige fat is reduced with obesity, and the challenge is to prevent its loss with ageing or to reactivate existing depots or both. This is difficult, as the current gold standard for assessing BAT function in humans measures radio-labelled glucose uptake in the fasted state and is usually dependent on cold exposure and the same subject can be found to exhibit both positive and negative scans with repeated scanning. Rodent studies have identified multiple pathways that may modulate brown/beige fat function, but their direct relevance to humans is constrained, as these studies typically are undertaken in cool-adapted animals. BAT remains a challenging organ to study in humans and is able to swiftly adapt to changes in the thermal environment and thus enable rapid changes in heat production and glucose oxidation.

## Introduction

The subject of brown adipose tissue (BAT) has become increasingly topical and controversial since its re-discovery in adult humans in 2007 ^1^. The simultaneous publication of three studies in the *New England Journal of Medicine* demonstrating the unequivocal detection of brown fat in adult humans ^2– 4^ paved the way for an exponential rise in publications on this subject ^5^. Brown fat is important because, though present in relatively small amounts in the body, it has the potential to rapidly produce large amounts of heat and thus impact on both energy balance and glucose and lipid homeostasis ^6, 7^. This is exemplified in the rapid activation of brown fat around the time of birth and the critical role it plays in the prevention of hypothermia ^8^. The “re-discovery” of brown fat has been accompanied by the identification of beige adipocytes (that is, small clusters of brown-like white adipocytes within white fat depots) ^9, 10^. Furthermore, lineage-tracing experiments in mice indicated that classic brown adipocytes, characterised as possessing the unique mitochondrial uncoupling protein 1 (UCP1), have a common lineage with skeletal muscle and are very different from the cellular origins of beige and white adipocytes ^8, 11^.

**Figure 1.**
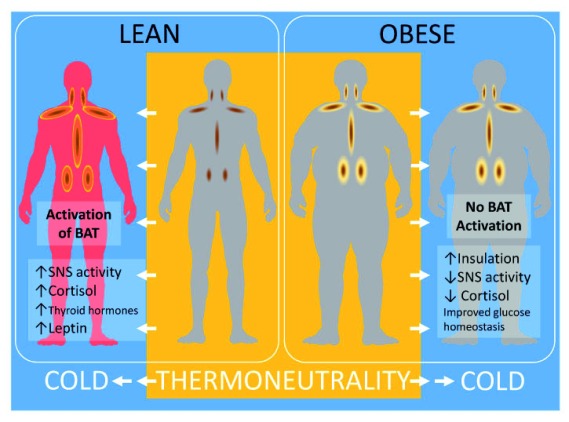
Summary of the impact of obesity on brown adipose tissue function and regulation in adult humans and the potential benefits of chronic cold exposure. ATGL, adipose triglyceride lipase; BAT, brown adipose tissue; FFA, free fatty acid; HSL, hormone-sensitive lipase; MGL, monoglyceride lipase; NEFA, non-esterified fatty acid; SNS, sympathetic nervous system; T, temperature; TG, triglyceride; WAT, white adipose tissue.

When UCP1 is stimulated, usually by the sympathetic nervous system, this results in the free flow of protons across the inner mitochondrial membrane ^12^, thereby bypassing the need to convert ADP to ATP, as occurs in the mitochondria of all other tissues. The primary stimulus for uncoupling remains contentious but is considered to be the release of fatty acids from lipid either within or surrounding brown adipocytes ^13^. Brown fat has the potential to produce far more heat per unit mass than any other organ in the body ^8^. Furthermore, the amount of UCP1 in classic brown fat is 10 times greater than that in the beige fat of rodents ^14^, meaning that the latter has a much smaller capacity to impact on whole-body energy balance. Beige fat, however, has the largest potential as a therapeutic target in the prevention of obesity or diabetes (or both) because it can be present in many white depots as clusters of pre-adipocytes that then can be recruited ^14^. However, the capacity of beige fat to modulate metabolism (especially glucose oxidation) may be mediated in part by mechanisms that do not involve UCP1 and have been proposed to be non-canonical ^15^. As summarised in Figure 1, the amount of activity of brown fat is reduced with raised white fat mass in obesity and its accompanying metabolically compromised endocrine environment.

## Advances in our understanding of the amount and activity of brown fat in adult humans

The gold standard by which the activity of brown fat is assessed in adult humans is still positron emission tomography-computed tomography (PET-CT) ^16^. This was the original method used to identify brown fat, and the same technique is used clinically around the world. It is a method that identifies brown fat from the uptake of radio-labelled glucose (fludeoxyglucose, or ^18^FDG) and is measured relative to the amount of glucose uptake in other tissues ^16^. However, to gain a significant signal within brown fat, the subject needs to be both fasted and cold-exposed ^17^. A better tracer than glucose for assessing brown fat thermogenesis is acetate ^18^, which is converted into acetyl-CoA within the cell and then incorporated into components of the citric acid cycle (a reaction that does not occur for ^18^FDG). Consequently, as brown fat rapidly turns over, the radio-labelled carbons in acetate are released as carbon dioxide and the amount of positron label lost is directly proportional to the metabolic activity of the tissue ^19^, which in the case of brown fat is substantial ^18^. When brown fat is activated by cold exposure, lipids stored within the depot are mobilised and oxidised to release heat ^19^. Acetate, however, is rarely used as a tracer, as it needs to be freshly prepared and, as it is not routinely used for cancer detection, such a facility is seldom available. This technical challenge means that our understanding of brown fat metabolism in humans remains constrained.

## Quantification of brown fat in humans

It is now recognised that the amount of brown fat is under-assessed in most studies which use radio-labelled glucose in PET-CT and varies considerably between individuals, with current estimates now ranging from about 30 to 350 mL in healthy subjects ^20, 21^. PET-CT studies examining the impact of an intervention on brown fat function in humans have to subdivide participants into brown fat “positive/+ve” and “negative/−ve” sub-groups ^22^ or only study BAT+ve individuals ^23^. This could be considered a rather arbitrary classification, as all adults have the potential to exhibit a brown fat+ve response when repeatedly assessed with PET-CT ^21^. However, one small study has shown that a BAT+ve scan is associated with greater UCP1 within supraclavicular brown fat ^24^. The same study demonstrated a large increase in UCP1 gene expression with cold exposure, although there was appreciable variation in response between individuals. Taken together, these findings indicate the rapid response of brown fat to cold thermal stimulation for which increased gene expression ^24^ could be a longer-term response that parallels the pronounced change in substrate uptake, compared with warm conditions, as recently indicated in human supraclavicular brown fat ^23^. It has also been suggested, from PET-CT studies, that more brown fat is present in females than in males, although these results are more likely to reflect the greater sensitivity to cold by females ^6^.

The practical and health limitations of using PET-CT, which involves significant exposure to radiation, prevent its widespread use on healthy populations, meaning other methods of assessing brown fat function *in vivo* are required. These include thermal imaging for which a close correlation between brown fat function, as measured with PET-CT, has been established ^25^. Furthermore, there are currently no reports of any individuals undergoing thermal imaging who do not have a hot spot that co-locates with the supraclavicular depot (that then increases in temperature with mild hand cooling) ^17^. Thermal imaging has also demonstrated a marked responsiveness of supraclavicular brown fat to diet ^26^ and its contribution to dietary-induced thermogenesis. It is therefore able to provide novel insights into the impact of diet on brown fat that cannot be readily obtained from PET-CT studies.

## Primary activators of brown fat

Reduced ambient temperature in humans remains the most potent stimulator of brown fat ^27^ and is not unexpected given that cold exposure to the extra-uterine environment is the primary catalyst for the onset of non-shivering thermogenesis at birth ^8^. This adaptation is accompanied by a rapid rise in a range of metabolic hormones, including catecholamines, thyroid hormones, cortisol, and leptin ^7^, with leptin co-locating onto the nucleus during cold induction of beige adipocytes ^28^. The extent to which repeated cold challenges can be used to enhance brown fat function in adults remains an important milestone for current research.

Importantly, glucose uptake in supraclavicular brown fat increases substantially with cold exposure and is positively correlated with the magnitude of cold-induced thermogenesis ^23^. Even in subjects who are classified as BAT−ve, an increase in mean glucose uptake is seen with cooling, although it is about 50% less than in those participants who are BAT+ve ^24^. A comparable adaptation has been observed in individuals who are diabetic, in whom the only study conducted to date indicates an improvement in glucose homeostasis ^29^. This was not fully explained, however, by the increase in glucose uptake within brown fat as measured by PET-CT ^29^. These findings are important given the potentially large amounts of brownable fat (up to 1,500 mL) as indicated in young healthy obese adults ^30^.

The exact amount of glucose oxidised by brown fat remains to be fully established, and it has been conservatively calculated that 300 mL of brown fat could dispose of at least 9 g of glucose per day ^20^. This value could be much greater on the basis of the threefold to fivefold increase in glucose uptake recently measured in supraclavicular fat with moderate cold exposure ^23^. With the innovative developments in studying brown fat mitochondria, further refinements in these calculations are likely. This is because two types of mitochondria have now been identified within rodent interscapular brown fat—the peridroplet and cytoplasmic domains—the latter of which regulates lipid supply, whereas the cytoplasmic domain could be more important in regulating glucose oxidation ^31^. Further adaptations as shown in beige fat of mice following cold exposure include the appearance of dense intra-adipose sympathetic arborisations ^32^, but have yet to be confirmed in humans. The capacity to predict which experimental models of browning are most relevant to humans could be further enhanced by the systematic integration of transcriptional profiles using online resources that are now being developed ^33^. The full extent to which changes in brown fat function can contribute to inadequate glucose homeostasis remains to be fully explored. It is noteworthy, however, that two recent studies have indicated that raised temperature is associated with increased risk of diabetes, either during pregnancy in Canada ^34^ or in all adults across the United States ^35^. Taken together, these findings emphasise the impact of climate change on human health and the extent to which adverse effects may be dependent on the body’s natural heat generator (that is, brown fat) ^5^.

Other targeted approaches to stimulate brown fat in humans have included the use of the β3-adrenoreceptor antagonist mirabegron (at a relatively high dose of 200 mg). Mirabegron promotes glucose uptake in a wide range of brown fat depots ^36^, as determined with PET-CT, as well as stimulating metabolic rate, although these two measures were not well correlated. Thus, it is possible that the effects of mirabegron are related more to effects on glucose metabolism in brown fat ^37^ rather than to heat production per se, but this needs further studying. Surprisingly, despite being published nearly 4 years ago, this study remains the only one of its kind. Both acute and chronic stimulation of the stress-sympathetic nervous system would appear to offer a route by which brown fat activity can be enhanced. For example, 24-hour infusion of hydrocortisone in adult males increases the temperature of the supraclavicular depot that co-locates with brown fat ^26^. In children, the stress associated with severe burn injury promotes the appearance of UCP1 in subcutaneous fat after 4 weeks ^38^.

## Limitation on our understanding of regulation of brown fat metabolism from rodent studies

The past 5 years have seen a prolific amount of research into brown fat, which suggests that it may have the capacity to improve metabolic homeostasis in adults, but such a goal will not be a straightforward outcome to achieve. This is, in part, because of the very different metabolic roles for brown fat in rodents and humans together with the divergence in experimental protocols used in animal studies ^39^ that do not readily compare with the human situation ^5^. However, the potential for UCP1 to generate heat appears to be similar between species ^40^, although the exact range of mechanisms involved remains to be fully established ^13^. It should be noted that laboratory rodents are typically maintained within a highly artificial environment, are usually fed a highly processed diet that is the same each day, experience no change in photoperiod (that is, fixed 12-hour day and 12-hour night) or ambient temperature, and have limited (if any) exposure to pathogens ^41^. Furthermore, the main brown (or beige) fat depot in adult humans is located within the supraclavicular region ^42^, and although this is also present in rodents ^43^, it has seldom been examined, as the interscapular and inguinal depots are primarily investigated.

Thus, it is important that the results from the plethora of rodent-based investigations are considered in light of the depot examined and the relevance to human adipose tissue of the identified pathways.

Although the focus of most rodent studies has been on identifying novel pathways that could be targeted to promote brown fat function, these have had relatively little impact on enabling sustainable interventions in adult humans. This could be for a number of reasons that now are starting to gain more consideration across the scientific community. The main concern is the thermal environment in which rodents are maintained when brown fat is examined, as it is clear that 20–21 °C represents a substantial thermal stress and that thermoneutrality is approximately 28–30 °C ^39^. Moreover, many rodent studies go on to examine the effect of further exposure to what, for laboratory mice, would be extreme cold (that is, about 6 °C), which results in maximal brown fat activation. This is rather an abrupt challenge and without a gradual decline in temperature, as would occur in the wild, is an unphysiological adaptation. In terms of susceptibility to metabolic-related disease, this is best illustrated in a mouse model of non-alcoholic fatty liver disease (NAFLD). Housing at thermoneutrality exacerbates the magnitude of NAFLD as well as removing any difference between sexes ^44^. Not surprisingly, those mice housed at 30 °C are characterised as possessing less brown fat and have lower plasma corticosterone concentrations which, in humans, are known to positively impact on brown fat function in some ^26, 45^ but not all ^46^ studies. Furthermore, thermoneutral housing accelerates atherosclerosis through increased metabolic inflammation, which surprisingly is uncoupled from insulin resistance ^47^. The link among inflammation, obesity-induced insulin resistance, and atherosclerosis has been clear for decades ^48^. It is likely, therefore, that these results in rodents have been confounded by chronic mild-cold stress and that better modelling of human physiology, especially with regard to the role of brown fat in metabolic disease, will be needed in future. The critical role of temperature has been highlighted *in vitro* ^49^, under which conditions the appearance of UCP1 also appears to be dependent on reduced ambient temperature ^28^.

## Conclusions

The re-discovery of BAT in adults has led to the recognition that promoting its activity could be an effective new strategy to improve metabolic homeostasis in a sedentary world where obesity and diabetes are prevalent. Although to date most studies in humans have focused on promoting heat production in brown fat, given the recent findings on increased glucose metabolism, this could be a more promising area in future research.
